# A New National Association

**Published:** 1885-10-20

**Authors:** 


A NEW NATIONAL ASSOCIATION.—The New York Medical Journal advocates the formation of a new National Medical Association, suggesting that a "committee of representative men in the several large cities be chosen by a small committee; the body to meet and select an equal number of other eminent men." The objection (says the Detroit Lancet) to this is *a dozen of men* will control the appointments in their interests and those of their friends. That is just what occurs in every Medical Society or Association we have ever known. They commence beautifully, but sooner or later the cliques get control and hold it.

DR. BELLENGER, of Charleston, S. C., had the misfortune, recently, to get into an altercation with a negro man in that city, and was under the necessity of shooting the negro. The negro was Democratic in politics, and highly esteemed by the white citizens, yet it appears that he was under the influence of ardent spirits, and was advancing upon the doctor at the time of the shooting.

